# The Relationship between Teachers and Peers’ Motivational Climates, Needs Satisfaction, and Physical Education Grades: An AGT and SDT Approach

**DOI:** 10.3390/ijerph17176145

**Published:** 2020-08-24

**Authors:** Filipe Rodrigues, Diogo Monteiro, Diogo S. Teixeira, Luís Cid

**Affiliations:** 1 Sports Science School of Rio Maior, Polytechnique Institute of Santarém (ESDRM-IPSantarém), 2040-413 Rio Maior, Portugal; diogomonteiro@esdrm.ipsantarem.pt (D.M.); luiscid@esdrm.ipsantarem.pt (L.C.); 2 Life Quality Research Center (CIEQV), 2040-413 Rio Maior, Portugal; 3 Research Center in Sport, Health and Human Development (CIDESD), 5001-801 Vila Real, Portugal; 4 Faculty of Physical Education and Sport, University of Lusófona (UHLT/FEFD), 1749-024 Lisbon, Portugal; diogo.teixeira@ulusofona.pt; 5 Center for the Study of Human Performance (CIPER), 1495-751 Lisbon, Portugal

**Keywords:** learning climate, performance climate, basic needs, physical education, grades, classroom

## Abstract

Grounded in achievement goal theory and self-determination theory, this study aimed to examine the associations of students’ perception of learning- and performance-oriented climates created by teachers and peers on basic psychological needs satisfaction, and consequently their relationships with physical education grades. This study had a cross-sectional design, and 589 students aged between 10 and 18 years (M = 12.93; SD = 1.49) were recruited for analysis. Participants completed a multisection survey assessing motivational climates and basic psychological needs, and physical education grades were provided by the physical education teacher. Students’ perception of learning-oriented climates created by teachers and peers was positively and significantly associated with basic psychological needs satisfaction. Additionally, these learning-oriented climates presented a significant indirect association with physical education grades. On the other hand, the performance-oriented climate created by teachers had a negative and significant relationship with basic psychological needs satisfaction and displayed a negative indirect relationship with physical education grades. The performance-oriented climate created by peers had a positive and significant relationship with basic psychological needs satisfaction and displayed a positive indirect relationship with physical education grades. The perception of performance-oriented climates created by peers could represent a boost within the students in physical education features. Teachers have the responsibility of promoting task and learning environments in which students experience positive outcomes, not only on a motivational level but also on a cognitive level.

## 1. Introduction

Physical education (PE) classes are able to promote enriching experiences and challenges among students. At the same time, they can be perceived as an unpleasant activity to others. Thus, motivation may explain these differences in PE participation, considering that the concept of human motivation is associated with how others influence human behavior [1]. To date, research on the PE setting has mainly examined students’ perception of motivational climates created by teachers as predictors of intentions towards physical activity [2] and emotional outcomes such as enjoyment [3]. However, there is scarcity of research on how peers’ motivational criterion for success and failure is associated with several behavioral and cognitive outcomes [4]. As evidenced by previous literature [5], the motivational climate created by peers can impact in a similar way to motivational climate created by the teacher. Hence, it seems of upmost importance to examine whether the possible differences between students’ perception of motivational climates created by teachers and peers are related to motivational determinants and, consequently, how students engage in PE classes.

### 1.1. Theoretical Frameworks in Physical Education Settings

When considering what features of PE teacher behavior hold importance for students’ motivation, two current theoretical frameworks provide clear direction on assessing behavioral outcomes. One of them is achievement goal theory (AGT) [6], more specifically, works from Ames [5] in the PE settings. AGT draws its basis from the motivational and achievement-related implications of differences in how students perceive or define success, based on their interaction with goal orientations. Learning-oriented students are prone for mastery, knowledge, and oriented for improvements [6]. On the other hand, performance-oriented students judge their competence based on comparison with others, placing emphasis on winning and ultimately on results [7].

Another line of research has considered how goal orientations are manifested at the situational level, created by the social context. Motivational climates are the psychological environments that are created by significant others (e.g., parents, peers, teachers, coaches) by designing conditions which provide feedback that is oriented to specific outcomes. Specifically, there are two contrasting motivational climates that have been discussed in previous literature [6]. Mastery or learning-oriented climates are when the situational features of teachers or peers regard the support of effort and emphasis on cooperation and team development [8]. An ego- or performance-oriented climate created by significant others is related to the focus on mastering the task at hand at any cost, and consists of normative evaluation and comparison with others [9].

Motivational climates as means of understanding achievement motivation in the PE context have been widely recognized by scholars [2,4,10]. Past literature generally provide support for positive associations between learning climates, intrinsic motivation, and intentions towards leisure-time physical activity [11], class involvement [4], and enjoyment [12] in the PE context. Previous AGT-based research also points to the negative consequences of marked performance climates in PE participation, such as less enjoyment, cognitive anxiety, and concentration disruption [13].

Teachers and peers, by creating a particular climate (i.e., learning or performance), may influence how students perceive their achievement goals [8,9]. However, students do not acknowledge their success solely based on situational factors. Specifically, the other contemporary framework that can be used to understand and augment understanding of students’ motivation and behavioral-related outcomes is self-determination theory (SDT) [1]. SDT describes the requirement of basic psychological needs (BPN) as determinants for optimal functioning [14]. Thus, students’ feeling of autonomy (i.e., feelings of volitional choice), competence (i.e., learning new skills and increasing capacities), and relatedness (i.e., connecting with others and creating social bounds) are results of motivational [2,15,16], emotional [11], and behavioral [4] outcomes. Hence, needs satisfaction is thought to be essential to nourish adaptive outcomes in PE involvement.

### 1.2. An Integrated Model of AGT and SDT in Physical Education Settings

From an SDT perspective, social factors such as need-supportive and need-thwarting behaviors provided from the social environment (e.g., teachers, coaches, trainers, peers) are responsible for how needs are met [1,14]. Considering that the social environment is situational in its nature, there could exist and interdependency between the different features of motivational climate that are highlighted by AGT in a similar manner as interpersonal behaviors. In fact, Duda, Appleton, Stebbings, and Balaguer [17] proposed a conceptualization that unites key tenets of motivational climates from the AGT and SDT frameworks. Specifically, the model considers the role of BPN satisfaction based on SDT as mediator in the relationships between motivational climates proposed by AGT and cognitive and behavioral outcomes. Previous literature has shown that learning- or task-oriented climates created by significant others are positively associated with needs satisfaction, whereas perceptions of a performance or an ego-oriented climate created by significant others is negatively associated with needs satisfaction [18].

Other empirical studies have provided further support of the association between the motivational climates grounded in AGT and different types of outcomes, considering the role of needs as mediators [2,19]. As previously stated, the factors which satisfy the needs for autonomy, competence, and relatedness promote adaptive outcomes and optimal functioning. Previous literature argues that high learning climates can fulfill these needs, and, therefore, can create conditions for positive results related to achievement and personal success [2,4]. In contrast, high levels of performance-oriented climates, especially coupled with low experience of needs satisfaction, are related with negative consequences such as goal-related frustrations and boredom [19,20]. This integrated model proposed by Duda and colleagues [17] also recognizes key features of individuals’ judgment and their level of BPN satisfaction, as well as context-specific outcomes. That is, a tendency to be learning- or mastery-oriented would positively predict BPN, while being performance-oriented would have the opposite results. This statement has been tested recently [4], opening new avenues to explore regarding the assessment of motivation in the PE context. However, the assessment of teachers’ and peers’ motivational climates in relation to students’ motivation is limited [21], and is particularly scarce in the context of PE engagement [4,8].

Even though past literature has advanced in regard to motivational climate assessment in the PE context, it seems that most research has focused its attention on the teacher-oriented climate [2,22,23], ignoring students’ perception of motivational climates created by peers to be learning-oriented or performance-oriented. As reported by Papaioannou [8,9], children interpret the same environment or context differently based on their previous experiences, personality differences, or behaviors exhibited by teachers and peers. Hence, how students perceive peers’ engagement in regard to achievement is different from how they perceive teachers’ motivational climates. Hence, the assessment of both students’ perception of motivational climates created by teachers and students seems of upmost importance.

Due to the limited literature comparing the influence of teacher and peers’ motivational climate on needs satisfaction and academic success (e.g., PE grades), several authors have called for more research on the assessment of goal achievement in relation to behavioral and cognitive outcomes [4,24,25]. Understanding how motivational climates from teachers and peers are associated with needs satisfaction and PE grades could give teachers crucial tools on how to promote and educate students to be supportive and task-initiative peers.

### 1.3. The Relationship between Motivational Climates, Needs Satisfaction, and Academic Performance

The analysis of motivational climates and academic performance in the school context has become a topic of interest in the educational community [2]. In fact, among the difficult conditions of the classroom context, student motivation is highlighted as a key variable when measuring academic achievement [26]. Recent studies have shown a significant association between motivational climates and academic success, specifically PE grades, considering the mediation role of motivational determinants [26,27]. This association could be related to the fact that students experiencing autonomy, competence, and relatedness satisfaction are more intrinsically motivated to put effort into academic tasks, increasing the likelihood of performing better in school classes [2,28]. A systematic review conducted by Taylor et al. [28] showed a positive and significant association between academic success (e.g., final exam grade, national test score, report card grades, etc.) and more self-determined motivation. These results are also supported by a study conducted by León, Núñez, and Liew [29], where motivated students presented higher grades in mathematics. Thus, bearing in mind that learning-oriented climates have a positive relationship between basic psychological needs, and that needs consequently are associated with positive outcomes such as academic success, it is theoretically possible that motivational climates could display an indirect association with PE grades. However, to our knowledge, studies considering the association between learning- and performance-oriented climates on grades are scarce, specifically considering the mediation role of autonomy, competence, and relatedness [26]. In addition, there are virtually no studies assessing both motivational climates oriented by PE teachers and peers and their relationship with PE grades. We address this research gap by testing the hypothesis that there is a positive association between learning-oriented climates created by PE teachers and peers and PE achievement [2,4,8].

### 1.4. Current Research

Recent attention has been devoted to the assessment and understanding of the motivational climates created by peers [2,4]. In fact, most of the studies have only focused on the learning- and performance-oriented climates created by teachers and their associations among adaptive outcomes [19]. However, researchers should assess not only the teachers’ perspective, but also the peers’ perspective, as they could lead to differentiated consequences. The research comparing the motivational processes from teachers and peers allows professionals and scholars to understand which factors contribute more to the academic success among students.

This study aimed to analyze the associations of motivational learning- and performance-oriented climates created by teachers and peers on PE grades, considering needs satisfaction as a mediator in this relationship. Based on past literature examining the role of motivational climates and needs satisfaction, it is hypothesized that: (a) learning-oriented climates created by teachers and peers have a positive association with needs satisfaction [19,22]; (b) performance-oriented climates created by teachers and students have a negative relationship with needs satisfaction [4]; (c) needs satisfaction is positively and significantly related with PE grades [2,4,19]; and, (d) motivational climates could have an indirect association with PE grades through the satisfaction of needs [2,19].

## 2. Materials and Methods

### 2.1. Participants and Procedures

This study had a cross-sectional design, considering the data from a convenience sample of 589 students (female = 390) aged between 10 and 18 years (M = 12.93; SD = 1.49). Participants were from 48 classes taught by 10 different PE teachers, and participants were recruited by convenience from two schools in the center region of Portugal. All students participated in regular PE classes two times per week according to the Portuguese educational system.

Data collection procedures were conducted in accordance with the 1964 Helsinki declaration and its later amendments or comparable ethical standards. Ethical approval was obtained by the Ethical Committee (reference number: UID04045/2020) prior to data collection. Afterwards, school boards and principals were contacted, research purposes were explained, and agreement was attained. Participants and their parents or legal guardians gave approbation, and parents or legal guardians signed informed consent prior to data collection. Questionnaires were provided by the researchers, and students in each class responded to the questionnaire in a classroom setting; however, their respective PE teacher was not present. Data were collected at the end of the year prior to release of grades. Time to complete the survey was approximately 15 min. Students received no counterpart but were thanked for their contribution.

### 2.2. Measures

Students completed the Learning and Performance Orientation in Physical Education Classes Questionnaire [8,9] adapted to the Portuguese context (the validation of the scale is currently under analysis). This adapted scale comprises 22-item split into four dimensions, namely: learning-oriented climates created by peers (5 items; item example “…My colleagues are very satisfied when I learn something new”), performance-oriented climates created by peers (5 items; item example “…My colleagues try to gain rewards by outperforming me”), learning-oriented climates created by PE teacher (6 items; item example “…The teacher looks most satisfied when every student learns something new”), and performance-oriented climates created by PE teacher (6 items; item example “…The teacher looks completely satisfied with those students who manage to win with little effort”). All items are followed by the sentence: “During physical education…” and students responded to each item using a 5-point scale anchored from 1 (“totally disagree”) to 5 (“totally agree”). The measurement model of the adapted Portuguese version in the present study displayed acceptable fit [χ^2^ = 894.324 (203); *p* < 0.001; CFI = 0.917; TLI = 0.903; SRMR = 0.048; RMSEA = 0.049 (0.044, 0.054)].

The Basic Psychological Needs Exercise Scale, adapted to the Portuguese classroom context [30] was used to measure autonomy (4 items; item example “I feel that I have the opportunity to choose how I do physical education activities”), competence (4 items; item example “I feel that I do physical education activities very well”), and relatedness satisfaction (4 items; item example “I feel comfortable with my classmates”). Students responded using a 5-point scale ranging from 1 (“totally disagree”) to 5 (“totally agree”). Composite score (i.e., needs satisfaction) was calculated using procedures reported elsewhere [31]. Specifically, we used a second-order approach to model each need as a composite score for needs satisfaction as a latent variable. The measurement model of this scale in the current study provided adequate fit: [χ^2^ = 275.208 (51); *p* < 0.001; CFI = 0.946; TLI = 0.900; SRMR = 0.042; RMSEA = 0.053 (0.050, 0.057)].

PE grades were obtained by requesting them directly from the PE teacher. The PE grade was obtained by averaging the mean scores of the academic year. Each grade was measured according to a 5-point coding system (1 = failure; 2 = poor; 3 = fair; 4 = good; 5 = excellent). Grades are a reliable measure of students’ involvement in PE settings [4,19].

### 2.3. Statistical Analysis

Descriptive statistics as well as bivariate correlation coefficients were calculated for each variable using IBM SPSS STATISTICS v23 [32]. Cutoffs for normality were considered based on guidelines [33], accepting scores within −2/+2 and −7/+7 for skewness and kurtosis, respectively. Composite reliability (CR) was calculated using Raykov formula, considering coefficients above 0.70 as acceptable [34].

Afterwards, a structural equation model considering all variables under analysis was performed in Mplus 7 [35] using the maximum likelihood robust estimator. The constructs (i.e., learning-oriented and performance-oriented climates created by teachers and peers, and needs satisfaction) were treated as latent variables, considering the respective number of items previously reported. For structural model assessment, several traditional and incremental indexes were considered, namely: comparative fit index (CFI), Tucker–Lewis index (TLI), standardized root mean square residual (SRMR), and root mean square error of approximation (RMSEA) and its respective 90% confidence interval (CI90%). Scores of CFI and TLI ≥ 0.90, and SRMR and RMSEA ≤ 0.80 were indicative of acceptable fit, as proposed by several authors [36,37,38]. It is worth to mention that the full information maximum likelihood (FIML) was used to handle the small amount of missing data at the item level (missing at random = 5%).

Direct and indirect effects were analyzed according to standardized coefficients and their respective 95% confidence interval (CI95%). Regression paths were considered significant if the CI95% did not include zero [39].

## 3. Results

### 3.1. Preliminary Results

Means for perceived learning-oriented climates created by teachers and peers were higher than those regarding performance-oriented climates. Skewness and kurtosis were contained within cutoffs, showing normal distribution. In addition, composite reliability coefficients were above acceptable, as shown in Table 1. The correlations displayed positive and significant associations between learning-oriented climates created by teachers and peers, needs satisfaction, and PE grades. Positive associations were found between performance-oriented climates created by peers, competence and autonomy satisfaction. Negative correlations were found between performance-oriented climates created by teachers, autonomy, competence, and relatedness satisfaction, and PE grades. Autonomy, competence, and relatedness satisfaction displayed a positive and significant association with PE grades.

### 3.2. Structural Model

**Figure 1:**
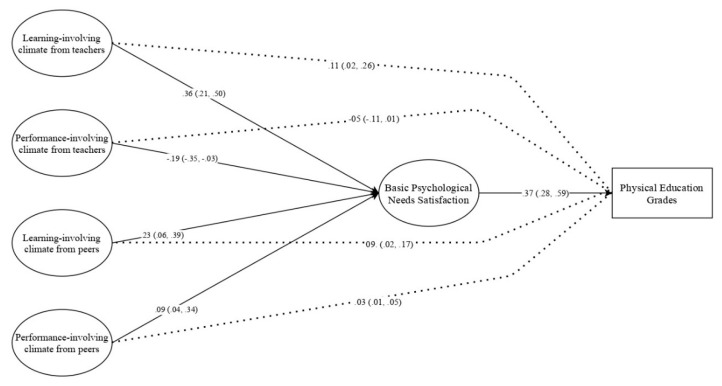
Structural model. Note: coefficients are standardized; brackets = confidence interval at 95%; dashed lines = indirect effect.

The structural model provided an adequate fit to the data: χ^2^(314) = 671.990; *p* < 0.001; CFI = 0.905; TLI = 0.901; SRMR = 0.067; RMSEA 0.047 [0.042, 0.052]. Hence, direct and indirect paths were analyzed (see Figure 1). Statistically significant direct paths were found as theoretically proposed, namely: (a) learning-oriented climates created by teacher and peers were positively associated with needs satisfaction; (b) the performance-oriented climate created by the PE teacher predicted negative and significant needs satisfaction; and, (c) needs satisfaction was a positive and significant predictor of PE grades. Regarding indirect effects, several results emerged, specifically: (a) learning-oriented climates created by teachers and peers had a positive and significant indirect association with PE grades; (b) performance-oriented climates created by PE teachers had a negative and significant indirect association with PE grades; and, (c) performance-oriented climates created by peers had a positive and significant indirect association with PE grades. The indirect associations between motivational climates and PE grades are mediated by the satisfaction of basic psychological needs, as seen in Figure 1.

**Figure 2:**
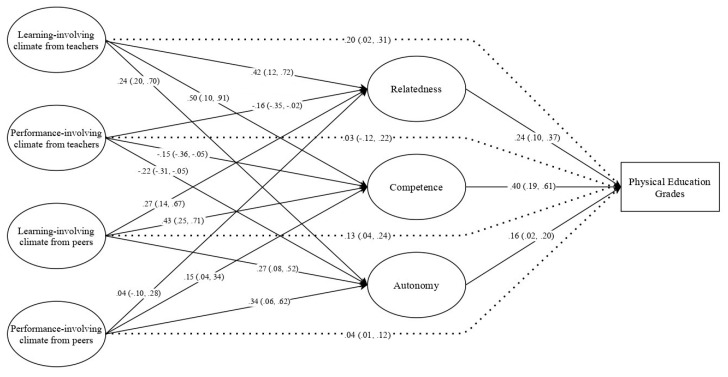
Exploratory structural model considering each need as independent mediator. Note: coefficients are standardized; brackets = confidence interval at 95%; dashed lines = indirect effect.

For exploratory purpose, we examined the structural model considering each need independently. The structural model displayed acceptable fit to the data: χ^2^(344) = 1464.732; *p* < 0.001; CFI = 0.915; TLI = 0.902; SRMR = 0.071; RMSEA 0.064 [0.061, 0.068]. Regarding direct paths, learning-oriented climates created by teachers and peers were positively and significantly associated with autonomy, competence, and relatedness satisfaction. Performance-oriented climates created by teachers displayed a negative and significant association with all needs. On the other hand, Performance-oriented climates created by peers showed a positive and significant relationship with competence and autonomy satisfaction, but not with relatedness satisfaction. Regarding indirect paths, several results emerged, specifically: (a) learning-oriented climates created by teachers and peers had a positive and significant association with PE grades; (b) performance-oriented climates created by PE teachers had a non-significant indirect association with PE grades; and, (c) performance-oriented climate created by peers had a positive and significant indirect effect on PE grades. For detailed information see Figure 2.

## 4. Discussion

With regard to past literature and agendas for future research, this study aimed to analyze the associations between students’ perception of learning- and performance-oriented climates created by teachers and peers and PE grades, considering autonomy, competence, and relatedness satisfaction as possible mediators. The current results provided new insights on how the perceptions of learning- and performance-oriented climates created by teacher and peers are distinct dimensions which have different relationships with needs satisfaction and PE grades. The results confirm our hypothesis that learning-oriented climates created by teachers and peers have a positive association with needs satisfaction. In addition, the current results also support partly our hypothesis that performance-oriented climates created by teachers and students have a negative relationship with needs satisfaction. Needs satisfaction was positively and significantly associated, as theoretically expected [2,4,19], and motivational climates showed an indirect association with PE grades through the satisfaction of needs, as hypothesized.

The present study found that perceptions of both learning-oriented climates created by teachers and peers displayed a positive association with students’ needs satisfaction. This is consistent with previous literature that analyzed the impact of social key agents (i.e., teachers, coaches) on the fulfillment of BPN proposed by the SDT framework in the PE context [2,19,20] as well as in the sport domain [17,40,41]. Additionally, in line with past works on the motivational impact of peers [4,21], the current study highlights that the level of BPN satisfaction in PE settings is associated not only with the motivational climate created by the teachers, but likewise by the motivational climates oriented by peers. That is, students’ perception of learning-oriented climates created by peers may have a similar association with needs satisfaction as the learning-oriented climate created by teachers. Hence, teachers encouraging students to choose different activities, making evaluations on individuals rather than as comparisons with others, providing feedback for improvement, and fostering feelings of being part of a group among students could represent an increase in needs satisfaction and provide students with the necessary tools to be supportive of each other. Regarding the learning motivational climate created by peers, for those who thrive when learning new skills, the connection shared among peers and the creation of an experience of autonomy could likewise represent ways of increasing needs satisfaction.

Learning-oriented climates created by teachers and peers displayed higher associations with competence satisfaction (β = 0.43–0.50), when compared to autonomy (β = 0.24–0.27) and relatedness (β = 0.27–0.42) satisfaction. Hence, Portuguese student seem to learn new skills and master other capacities when looking at teachers and peers as need-supportive figures when learn something new. These results are somewhat in line with previous studies [1,2], showing that motivational climates, specifically those oriented towards learning, are more relatable to competence satisfaction.

In agreement with the SDT perspective [1], needs satisfaction positively predicted PE grades, supporting findings from previous research [2,4,19]. Since the satisfaction of needs is related to positive outcomes, such as self-determined motivation [1,15,16], intentions towards physical activity [2], and enjoyment [3], it would be excepted that higher levels of autonomy, competence, and relatedness would impact higher academic success. Hence, those students who exercise volitional choice (e.g., choosing exercise combinations), who improve skills (e.g., learning how to dribble faster), and those who connect with peers (e.g., creating friendships during class) are able to engage and involve themselves more in PE classes, being positively associated with their academic grades, as seen in the present study.

As theoretically expected, motivational climates focused on the learning processes displayed a significant indirect association on PE grades. This aligns with the empirical viewpoint of past research [2,4,19,23] that suggests that one of the ways that motivational climates can influence positive outcomes is through the experience of needs satisfaction, channeling improvement, effort, self-referenced evaluation, and cooperation. Recognizing the mediation role of needs satisfaction between motivational climates and grades is of great importance, as shown by the indirect effect. That is, without the experience of autonomy, competence, and relatedness fulfillment, learning- and performance-oriented climates would not exert and association with PE grades. When students perceive their PE teacher to value being skillful and process-oriented, their volition to be more engaged in classes could be referenced by the high levels of autonomy, competence, and relatedness satisfaction. As a result, the satisfaction of each basic need may increase the components of academic engagement. By having a sense of autonomy, students’ needs are triggered, because they feel greater freedom to do their assignments, be participative in the activities they like the most, and have a sense of choice in the curricular activities [26,29]. By having their need of relatedness satisfied, students feel at ease and comfortable to express themselves in class and to relate with peers, which contributes to a positive engagement towards PE classes [2]. By having the need for competence satisfied, students experience mastery, which motivates them to invest extra effort in PE classes [19]. This is because task-oriented climates promote needs satisfaction and encourages initiative to be self-endorsed and self-determined, as previously stated [1]. Hence, teachers focusing on leaderships roles, giving private recognition, and emphasizing effort are able to promote positive outcomes [23,42].

Even though it was anticipated that there would be a positive association between learning-oriented climates and PE grades and a negative association between performance-oriented climates and PE grades, the performance-oriented climate created by peers had a positive and significant indirect effect on PE grades. Thus, it seems that peers’ goal normative and self-referenced criteria could have a positive effect on how students are evaluated during class and impact others during PE. In fact, as stated by Duda [17], being performance-oriented (or describe in sports as ego goal perspective) is not always clearly problematic. For example, young individuals who are very confident and skillful can demonstrate a high performance both at a physical and psychological level. Hence, learning- and performance-oriented climates in each student could positively impact behavioral outcomes, acting as orthogonal factors rather than dichotomously, as seen in previous studies [37,43]. Nevertheless, these are speculative references and should be tested in the PE context before drawing general conclusions.

### 4.1. Strengths, Limitations, and Agenda for Future Research

The current study has a number of strengths, including a comprehensive test of both motivational climates oriented by PE teacher and peers, an under-researched area in the assessment of achievement and motivation in the PE context. This research is also innovative as it explores the extent to which the effects of academic success could be attributed to the motivational climates created by PE teachers and peers, considering the role of needs satisfaction as composite score and as each factor independently. The strengths of the current research are the adoption and integration of the AGT and SDT in one model, an appropriate model that provides a clear set of predictions and associations with the motivational determinants of academic success; the use of valid measures as reported by the measurement models; and the assessment of an objective measured behavior, namely, PE grades. The strengths of the present study also include a focus on the motivational determinants of PE grades, a priority area of research since it has been reported that children and young adults are increasingly less physically active.

As with all research conducted to date, this study has its limitations of its own. First, due to the lack of balanced groups with distinct characteristics (e.g., age, gender), multigroup analysis could not be performed. In this regard, future studies should test current model between groups, since there could exist differences among students’ perceptions of motivational climates initiated by teachers and peers [8,9]. Second, data collection procedures were performed within a convenience sample of two schools with a similar educational system. Other factors such as cultural background could present different results [13]. The authors also acknowledge the use of self-reported measures as a limitation and propose future avenues of research using qualitative or mix-method data as in previous studies [10]. Interesting results could emerge by triangulating the student perception of motivation climates with those perceptions from other groups (e.g., researchers, parents). Thus, more studies are warranted to examine the implications of initiating learning and performance motivational climates on students’ academic success. Third, even though this study considered needs satisfaction as a possible mediator, forthcoming studies should consider a mediation analysis approach to examine the mediation role of needs or other motivational constructs proposed by the SDT framework (e.g., motivational regulations, motives) in the relationship between motivational climates and academic success. In addition, future studies should test the 2 × 2 model proposed by Elliot and McGregor [44] to examine the distinct relationship among learning- and performance-oriented approaches, the avoidance framework, and motivational and behavioral outcomes. Last, it is important to mention the cross-sectional design inherent in this study. As such, longitudinal research using an actual intervention protocol would be appropriate for assessing the effects of motivational climates at the dispositional and situational level on motivational tenets and behavioral outcomes. According to Warburton (2017), students’ perception of learning- and performance-oriented climates vary across time, leading to differentiated results.

### 4.2. Practical Implications

PE teachers should understand the importance of studying perception of their own as well as peers’ orientations towards achievement and how this impact other students. Thus, awareness that aims to enhance positive PE engagement (e.g., PE participation, grades, effort, self-efficacy) is needed. A model of motivation that integrates conceptualizations of achievement could be an important tool that promotes adaptive outcomes. Ames [5] proposed the task–authority–rewards–grouping–evaluation–time (TARGET) approach as a way to foster task and learning-oriented climates. The six factors that teachers have control over when they teach are as follows: (a) the task that they ask students to perform; (b) the amount of authority they allow students to have; (c) how and when students are rewarded; (d) the criteria of how students are grouped; (e) how students are evaluated accordingly; and (f) the amount of time needed to learn materials or tasks. Teachers may consider the TARGET approach to develop climates that are mainly learning-oriented, considering each aspect and cue inherent in the TARGET factors. In addition, assessing students’ orientation could give teacher crucial cues on how to conduct and schedule PE classes and thus improve academic success.

## 5. Conclusions

Overall, our hypotheses were supported by the current results. Learning-oriented climates displayed a positive and significant association with needs satisfaction, and an indirect relationship with PE grades, considering needs as mediators. Teachers are accountable for promoting task and learning-oriented environments in which students experience positive outcomes, not only on a motivational level but also on a behavioral level. The assessment task and learning-oriented behaviors of PE teachers and peers are paramount, and it is crucial to improve students’ involvement in PE, ensuring that the environment created by the teacher and peers is as healthy and flexible as possible. Within education, there is a pressing need for more theory-informed and evidence-based practice in PE settings. This study suggests that the current results should not only underpin work conducted with and by PE teachers, but should likewise enable other key social agents such as peers to create favorable environments to promote adaptive outcomes. These guidelines should be considered as healthy and optimal for PE engagement but also for other relevant day-to-day activities, such as out-of-school sport participation and leisure-time physical activity.

## Figures and Tables

**Table 1:** Descriptive statistics, composite reliability, and correlations.

Variables	M	SD	S	K	Correlations						
					1	2	3	4	5	6	7
1. LO climate created by Teachers	4.20	0.63	−0.40	0.27	*0.79*						
2. PO climate created by Teacher	3.52	0.86	−0.20	−0.17	0.08	*0.72*					
3. LO climate created by Peers	4.50	0.58	−0.69	−0.20	0.65 **	0.25 **	*0.81*				
4. PO climate created by Peers	2.39	0.91	0.33	−0.13	−0.16 *	0.33 **	−0.33 **	*0.72*			
5. Autonomy Satisfaction	3.30	0.72	−0.58	0.09	0.30 **	−0.24 *	0.38 **	0.34 **	*0.70*		
6. Competence Satisfaction	3.71	0.67	−0.37	0.08	0.49 **	−0.11 *	0.37 **	0.20 **	0.43 **	*0.87*	
7. Relatedness Satisfaction	4.15	0.78	−1.07	0.08	0.52 **	−0.74 *	0.48 **	−0.06	0.50 **	0.32 **	*0.78*
8. Physical Education Grades	3.38	0.66	0.12	0.04	0.28 **	0.08	0.30 **	−0.16 **	0.22 **	0.37 **	0.39 **

Note: LO = learning-oriented; PO = performance-oriented; M = mean, SD = standard deviation, S = skewness; K = kurtosis, in diagonal and italic = composite reliability coefficients; * *p* = 0.05; ** *p* = 0.01.
